# Jamb and Jamc Are Essential for Vertebrate Myocyte Fusion

**DOI:** 10.1371/journal.pbio.1001216

**Published:** 2011-12-13

**Authors:** Gareth T. Powell, Gavin J. Wright

**Affiliations:** Wellcome Trust Sanger Institute, Hinxton, Cambridge, United Kingdom; Monash University, Australia

## Abstract

Cellular fusion is required in the development of several tissues, including skeletal muscle. In vertebrates, this process is poorly understood and lacks an in vivo-validated cell surface heterophilic receptor pair that is necessary for fusion. Identification of essential cell surface interactions between fusing cells is an important step in elucidating the molecular mechanism of cellular fusion. We show here that the zebrafish orthologues of JAM-B and JAM-C receptors are essential for fusion of myocyte precursors to form syncytial muscle fibres. Both *jamb* and *jamc* are dynamically co-expressed in developing muscles and encode receptors that physically interact. Heritable mutations in either gene prevent myocyte fusion in vivo, resulting in an overabundance of mononuclear, but otherwise overtly normal, functional fast-twitch muscle fibres. Transplantation experiments show that the Jamb and Jamc receptors must interact between neighbouring cells (in trans) for fusion to occur. We also show that *jamc* is ectopically expressed in *prdm1a* mutant slow muscle precursors, which inappropriately fuse with other myocytes, suggesting that control of myocyte fusion through regulation of *jamc* expression has important implications for the growth and patterning of muscles. Our discovery of a receptor-ligand pair critical for fusion in vivo has important implications for understanding the molecular mechanisms responsible for myocyte fusion and its regulation in vertebrate myogenesis.

## Abstract 2

Jamb and Jamc are an essential cell surface receptor pair that interact to drive fusion between muscle precursor cells during zebrafish development.

## Author Summary

The fusion of precursor cells is a crucial step in many biological processes, one of which is the development of skeletal muscle. The molecular and cell biology of fusion of muscle precursors has been well described in *Drosophila melanogaster* larvae, leading to insights into the process in vertebrates. However, the identity and mechanism of action of essential cell surface proteins for fusion between vertebrate muscle precursors has previously been lacking. Here, we describe a vertebrate-specific cell surface receptor pair that is essential for fusion in zebrafish: Jamb and Jamc. Loss of function of either receptor causes a near-complete block in fusion, resulting in an overabundance of mononucleate muscle fibres that are otherwise overtly normal. We demonstrate that Jamb and Jamc physically interact and are co-expressed by muscle precursors. Moreover, we show that the interaction between them is essential for fusion between neighbouring precursors in an embryo. We hypothesise that binding of Jamb to Jamc is a necessary recognition and adhesion step permissive for, but not sufficient to cause, myocyte fusion. Knowledge of these molecular components in vertebrates will lead to better understanding of how fusion is controlled to pattern skeletal muscle tissue.

## Introduction

Cell–cell fusion is crucial for several biological processes, including placental development [1], bone remodelling [2], fertilisation [3], and formation of skeletal muscle fibres [4], but surprisingly remains poorly understood. Skeletal muscle forms the bulk of tissue in vertebrates and is composed of bundles of long syncytial fibres formed by the fusion of post-mitotic muscle precursor cells (myocytes). It is a highly regenerative tissue, constantly undergoing repair and growth through the fusion of myocytes to form new fibres or supplement established ones. Impairment of the function of muscle, through age or genetic lesion, results in mild-to-severe pathologies that shorten lifespan, reduce quality of life, and demand a high burden of care. A more complete understanding of the molecular mechanisms of muscle development may lead to better treatment of muscle diseases and greater insights into regenerative medicine.

Muscle development has been well characterised in the larval body wall musculature of *Drosophila melanogaster*, where fusion occurs between two sub-populations of myoblasts, referred to as the fusion-competent myoblasts (FCMs) and founder cells [5]–[7]. The process of fusion has been resolved into a series of intermediate steps through ultrastructural analysis [8]–[10] and identification of the molecular components through forward genetics screens [11]. A critical step in fusion is the initial recognition and adhesion between the two cell types. This is regulated by the mutually exclusive expression of the cell surface receptor proteins Kirre and Sns, which form a heterophilic receptor pair between neighbouring cells [12]–[17]. Mutations in genes encoding these cellular recognition receptors (and their partially redundant paralogues Rst and Hbs) result in a severe block in fusion between the muscle precursors. In vertebrates, a functionally equivalent heterophilic receptor pair that is essential for myocyte fusion in vivo has yet to be identified [18]. One approach to isolate the vertebrate receptors has focussed on using sequence orthology to the fruitfly proteins—a rationale which is validated by emerging evidence that the molecular pathways important for myocyte fusion are conserved across these species [19]–[23]. Cell culture experiments have also suggested the involvement of several other cell surface proteins in vertebrate myocyte fusion, for example BOC and CDO [24]. Loss-of-function studies of these candidates resulted in mild disruption of myocyte fusion in vivo, leading to the view that this process involves several partially redundant proteins in vertebrates [18]. Only one vertebrate receptor, Kirrel3l (originally named Kirrel), has been identified as essential for myocyte fusion in vivo by using an antisense morpholino to knockdown the protein in zebrafish embryos [22]. There is no known Kirrel3l counter-receptor involved in the process of fusion, suggesting that other important vertebrate receptors remain to be discovered.

In this study, we identify two vertebrate cell surface receptors that are crucial for myocyte fusion: Jamb and Jamc (official nomenclature: Jam2a and Jam3b, respectively; Entrez gene: 100005261, 569217, respectively). The mammalian orthologues of both genes, commonly referred to as JAM-B and JAM-C (after rationalisation of the gene family nomenclature within the field [25]), have well-characterized roles in leukocyte migration [26], tight junction formation [27]–[29], and spermatogenesis [30]. Mouse Jam-B (Jam2) and Jam-C (Jam3) are members of a small sub-group of immunoglobulin superfamily cell surface proteins that is restricted to the deuterostome lineage (TreeFam [31]). They contain two extracellular immunoglobulin superfamily domains, a single transmembrane domain and a short cytoplasmic domain ending in a type II PDZ domain binding motif [32]. Heterophilic interactions between Jam-B and Jam-C are thought to be important for leukocyte transmigration across vascular endothelia [26] and the polarisation of spermatids necessary for complete differentiation into functional spermatozoa [30], but to date, there is no reported function for Jam-B and Jam-C in muscle development.

We have shown here that *jamb* and *jamc* are co-expressed in developing myoblasts and, by using mutant zebrafish, demonstrate that the physical interaction between them is essential for myocyte fusion in vivo. By analysing the mutant phenotypes and showing that *jamc* expression is misregulated in a muscle patterning mutant, we provide new insights into the regulatory mechanisms that govern vertebrate myogenesis.

## Results

### Zebrafish *jamb* and *jamc* Are Co-Expressed by Fast Muscle Myoblasts

**Figure 1:**
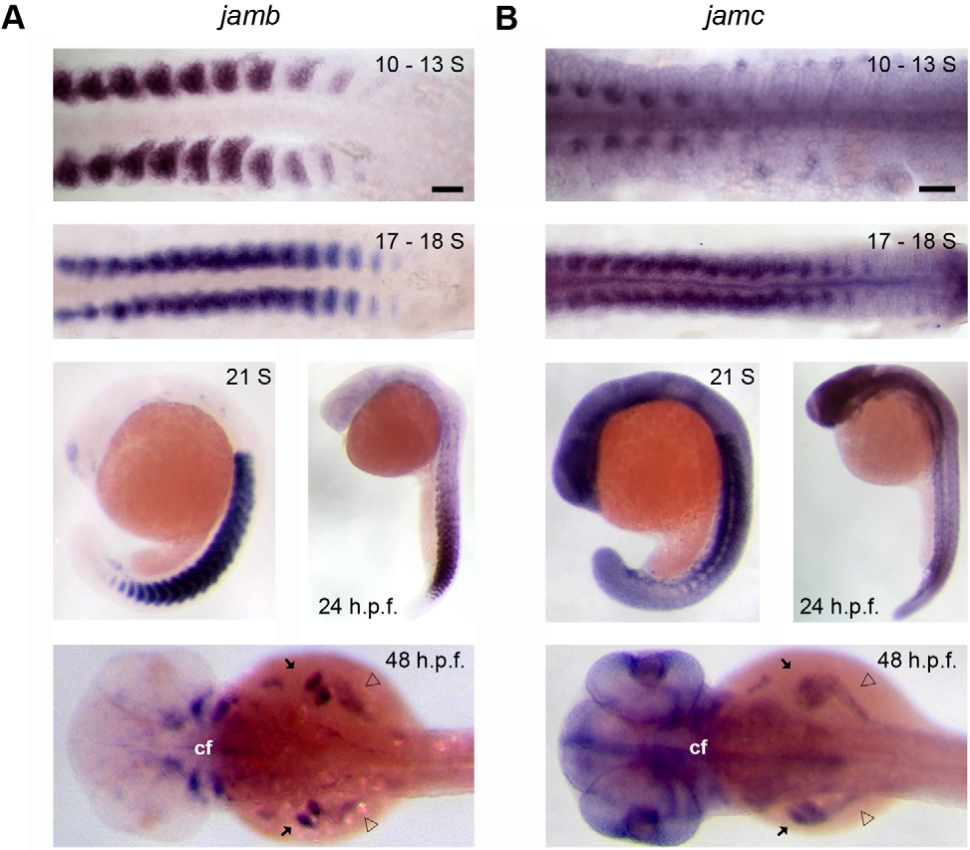
*jamb* and *jamc* are co-expressed in myoblasts. (A–B) Wholemount in situ hybridisation of *jamb* (A) and *jamc* (B) show expression in myoblasts during somitogenesis (10–13 somites, 17–18 somites, and 21 somites). Expression is attenuated in the myotome after the completion of primary myogenesis (24 h. p. f.) and then later upregulated in craniofacial (cf), pectoral fin (arrows), and hypaxial (open arrowheads) muscle mesoderm (48 h. p. f.). Scale bars represent 50 µm.

To identify novel receptor pairs that might be involved in myocyte fusion, we queried our large database of extracellular protein interactions constructed by screening a library of 249 zebrafish receptor proteins using the AVEXIS assay and supported by embryonic expression patterns of the corresponding genes during zebrafish embryonic development [33]–[35]. One pair, Jamb and Jamc, was selected because both genes are expressed by dividing myoblasts during primary myogenesis, but in distinct patterns. *jamb* is expressed by all fast muscle myoblasts shortly after the formation of each somite (Figure 1A). After approximately 10–13 somites have formed, *jamc* is initially highly expressed in a small, medial sub-population of fast muscle myoblasts along the dorsal-ventral axis (Figure 1B, 10–13 somites). Over time, the expression domain of *jamc* expands to include all myoblasts in the hypaxial and epaxial regions of the myotome (Figure 1B, 17–18 somites, 21 somites). *jamb* and *jamc* are co-expressed by all myoblasts in the anterior somites by the 17–18 somites stage (Figure 1, 17–18 somites) and in posterior somites at later stages (Figure 1, 21 somites). Whilst highly expressed in developing muscle, *jamc* also appears to be expressed at a basal level throughout the embryo—an observation that is replicated using a second riboprobe specific to the 3′ UTR of *jamc* (unpublished data). The expression of both genes in the myotome is attenuated in axial musculature by 24 h post-fertilisation (h. p. f.), but subsequently upregulated in later-developing craniofacial, abdominal, and pectoral fin muscles (Figure 1A,B). We conclude that both *jamb* and *jamc* are expressed in the somites of the embryo in a wave along the anterior-posterior axis. Within each somite, *jamc* expression begins medially and spreads laterally throughout the domain of *jamb*-expressing fast muscle myoblasts over time, so that both genes are co-expressed by myoblasts during the initial period of fusion between somitic precursors [20],[36]. Dynamic co-expression of the *jamb* and *jamc* genes in the developing musculature and later forming muscles suggested a role for the interaction between these two cell surface receptor proteins in myogenesis.

### *jamb* and *jamc* Are Essential for Myocyte Fusion

**Figure 2:**
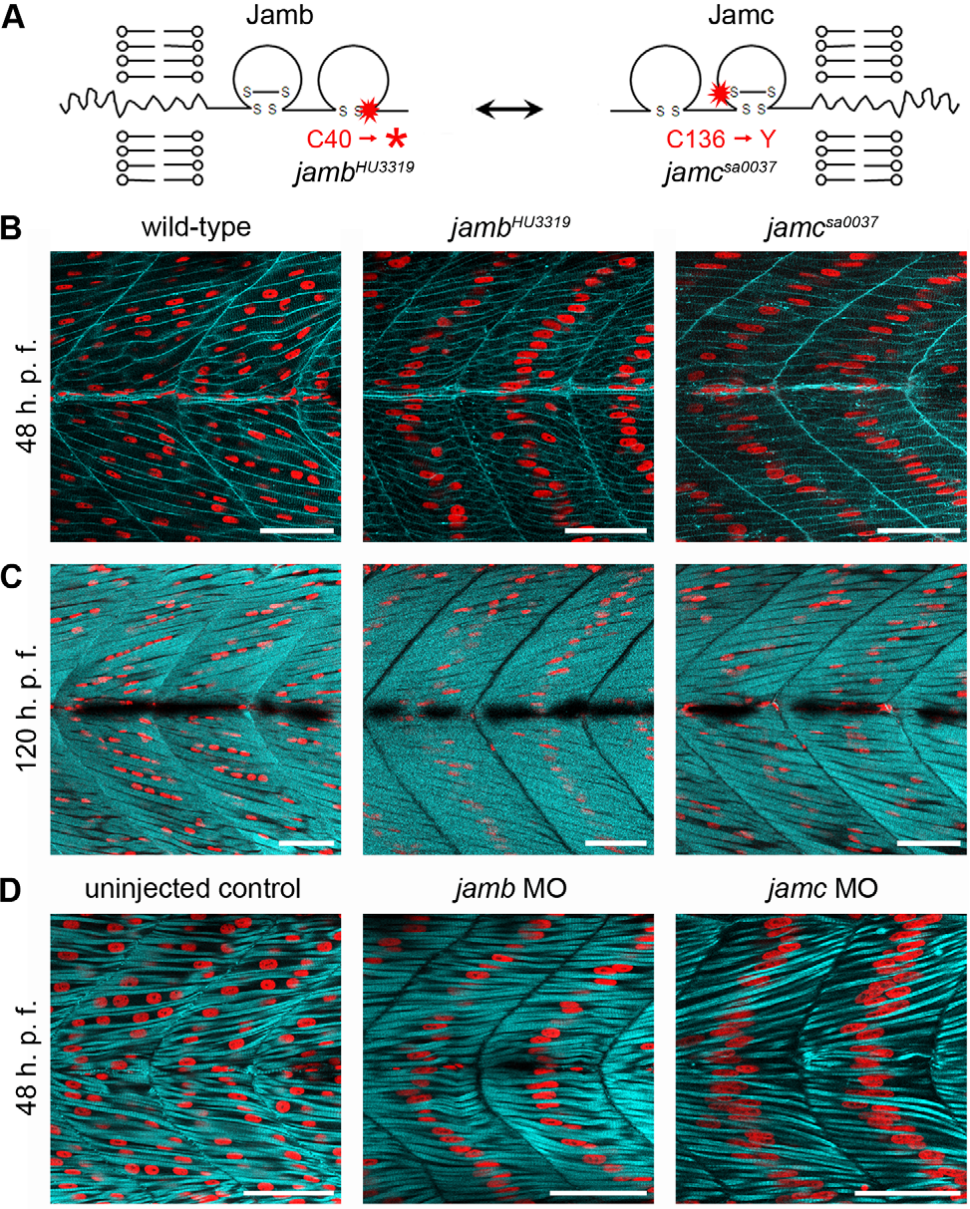
*jamb* and *jamc* are essential for myocyte fusion in vivo. (A) Schematics of Jamb and Jamc extracellular proteins. Red stars denote sites of mutation in *HU3319* and *sa0037* alleles. (B–C) Confocal microscopy images of fast-twitch muscle in wild-type, *jamb^HU3319^*, and *jamc^sa0037^* 48 h. p. f. (B) and 120 h. p. f. (C) Embryos labelled with membrane targeted RFP (mRFP, cyan; B) or phalloidin-Alexa488 (cyan; C) and DAPI (red) show overabundant, mononuclear myofibres in both mutants. (D) Confocal microscopy images of uninjected, *jamb*, and *jamc* translation-blocking morpholino-injected wild-type embryos, stained with DAPI (red) and phalloidin-Alexa488 (cyan) to stain F-actin in fast muscle fibres. The morpholino-injected embryos replicate the *jam* mutants' phenotype. Myotomes 12–13 shown, anterior left. Scale bars represent 50 µm.

To establish whether *jamb* and *jamc* were important for myocyte fusion in vivo, mutant alleles of both genes were obtained from the Hubrecht Institute (*HU3319*) and Sanger Institute Zebrafish Mutation Resource (*sa0037*; Figure 2A). Mutations within selected exons of *jamb* and *jamc* were identified by amplifying and directly sequencing PCR products from libraries of chemically mutagenised zebrafish. The *jamb^HU3319^* allele is a nonsense mutation that results in a premature stop codon near the N-terminus of the protein. A truncating mutation was not recovered for the *jamc* gene, but one allele, *jamc^sa0037^*, contained a missense point mutation in a cysteine residue (C^136^ to Y) that is predicted to form a structurally critical disulphide bond. Both *jamb^HU3319^* and *jamc^sa0037^* homozygous mutant embryos exhibited the same striking phenotype: regimented lines of centrally positioned nuclei within each myotome (Figure 2B). In wild-type embryos, somitic fast muscle myocytes fuse together to form multinucleate muscle fibres by approximately 24 h. p. f. [20],[36]. In *jamb^HU3319^* and *jamc^sa0037^* mutants, fast muscle myocytes did not fuse, but instead, fully elongated to form mononuclear fibres that spanned each somite by 48 h. p. f. (Figure 2B) and remained mononucleate until at least 5 days post-fertilisation (Figure 2C). We quantified the lack of fusion in subsequent transplant experiments: 95% and 85% of fast fibres remained mononucleate in *jamb^HU3319^* donor into *jamb^HU3319^* host and *jamc^sa0037^* donor into *jamc^sa0037^* host transplants, respectively (Table 1). To provide independent evidence that the mutations in both *jamb* and *jamc* were responsible for the phenotype, we injected translation-blocking morpholino antisense oligonucleotides targeted to both *jamb* and *jamc* into wild-type embryos. Embryos injected with either morpholino phenocopied the mutants, demonstrating that the phenotype was not due to closely linked mutations in either the *jamb^HU3319^* or *jamc^sa0037^* mutant lines (Figure 2D). From these experiments we conclude that zebrafish *jamb* and *jamc* are essential for myocyte fusion.

**Table 1:** Quantification of fused (multinucleated) and unfused (mononucleated) fluorescently labelled fast muscle fibres in transplanted hosts.

Donor Genotype	Host Genotype								
	Wild-Type			*jamb^HU3319^*			*jamc^sa0037^*		
	Unfused	Fused	*n* a	Unfused	Fused	*n*	Unfused	Fused	*n*
wild-type	3.1%	96.9%	254, 6	5.3%	94.7%	712, 7	8.4%	91.6%	403, 9
*jamb^HU3319^*	6.7%	93.3%	341, 8	94.9%	5.1%	526, 6	4.4%	95.6%	525, 9
*jamc^sa0037^*	71.3%	28.7%	630, 10	5.2%	94.8%	582, 7	85.3%	14.7%	218, 9
*jamb^HU3319^* and *jamc* MO^b^	87.8%	12.2%^c^	738, 16	n. d.			74.0%	26.0%	407, 9
*jamc^sa0037^* and *jamb* MO^b^	76.0%	24.0%^c^	250, 11	83.7%	16.3%	153, 6	n. d.		

a “n” denotes number of fibres, number of embryos analysed at 48 h. p. f. for each transplant experiment.

b Translation blocking morpholino injected into donor before transplantation. n. d., not determined.

c Minority fusion events are likely a result of incomplete morpholino knockdown, as seen in control transplants using *jamb^HU3319^* or *jamc^sa0037^* mutant hosts.

### Myogenesis Is Overtly Normal in Both *jamb* and *jamc* Mutants

**Figure 3:**
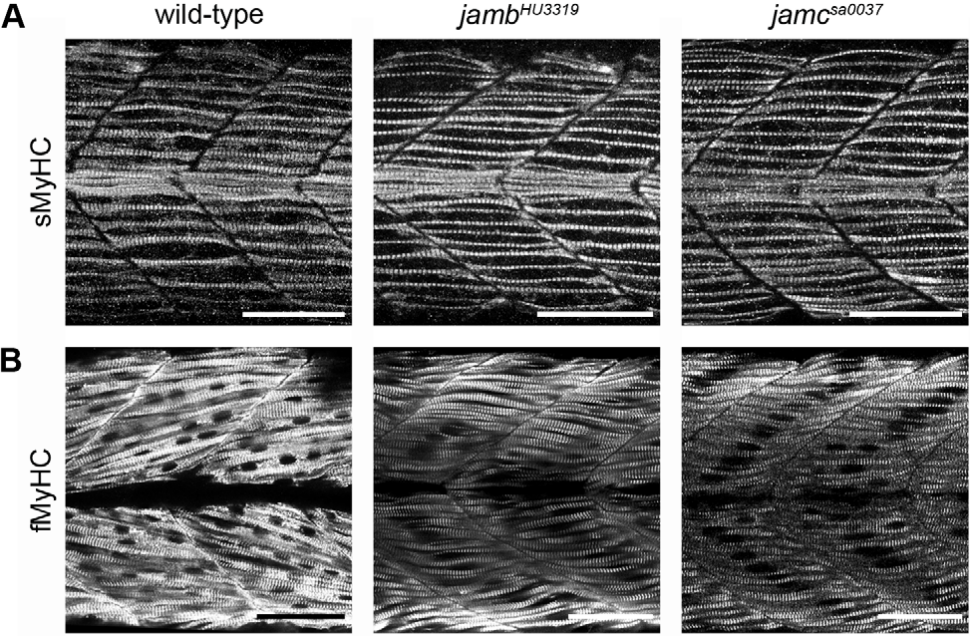
Fast and slow-twitch muscle fibres are correctly specified in *jamb^HU3319^* and *jamc^sa0037^* embryos. (A) Superimposed confocal microscopy images of 24 h. p. f. wild-type, *jamb^HU3319^*, and *jamc^sa0037^* embryos stained with monoclonal antibody F59, which detects slow-muscle-specific myosin heavy chain (sMyHC), show normal slow muscle development in both mutants. (B) Single optical sections of 48 h. p. f. wild-type, *jamb^HU3319^*, and *jamc^sa0037^* embryos stained with monoclonal antibody EB165, which detects fast-muscle-specific myosin heavy chain (fMyHC), show that the medial mononuclear fibres in both mutants have differentiated as fast-twitch muscle. Myotomes 12–13 shown, anterior left. Scale bars represent 50 µm.

In teleost fish, two spatially segregated muscle populations form during primary myogenesis: superficially located slow-twitch muscle and medial fast-twitch muscle [37]–[39]. Fast muscle fibres are syncytial (Figure 2B), but slow muscle fibres remain mononucleate during embryonic development [40]. To determine if the mononuclear muscle fibres in *jamb^HU3319^* and *jamc^sa0037^* mutants do correctly differentiate as fast-twitch muscle, we used antibodies that are specific for the slow and fast isoforms of myosin heavy chain (sMyHC and fMyHC). We observed that both mutants had the same number of normal, superficially positioned slow muscle fibres (Figure 3A) with no ectopic expression of sMyHC within the deeper fibres. The medially located, and more numerous, mononuclear fibres in both mutants expressed fMyHC (Figure 3B), suggesting that specification of fast-twitch muscle was unaffected. Finally, we observed no difference in the ability and timing of spontaneous twitching and response to tactile stimuli in either mutant relative to wild-type, suggesting that the muscles were innervated and fully functional (unpublished data). Together, these data suggest that both mutants are able to complete the myogenic programme, except for a specific defect in fusion. These findings also suggest that other aspects of terminal differentiation, such as elongation and sarcomerogenesis, do not depend upon myocyte fusion in vertebrates.

### Fast Muscle Fibres Are Supernumerary in *jamb* and *jamc* Mutants

**Figure 4:**
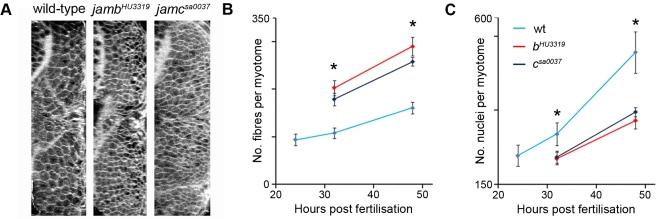
Fast muscle fibres are supernumary in *jamb^HU3319^* and *jamc^sa0037^* embryos. (A) Transverse sections projected from confocal microscopy images of 48 h. p. f. embryos labelled with mRFP. (B–C) Graphs showing a significant overabundance (1.6–1.8-fold) of muscle fibres in mutants compared to wild-type (B) and calculated muscle nuclei number in wild-type and mutant embryos between 24 and 48 h. p. f. (C). Asterisk denotes *p*≤0.001; one-tailed Student's *t* test; error bars represent standard deviation, see Tables S1 and S2 for number of embryos in each sample.

We observed an overt overabundance of fast muscle fibres in both mutants relative to wild-type embryos (Figure 2B). We quantified this increase by counting fibres outlined by a membrane-localised red fluorescent protein (mRFP) in optical cross-sections of wild-type and mutant embryos at 24, 32, and 48 h. p. f. (Figure 4A), revealing a statistically significant increase (*p*≤0.001) in fast fibre number in mutants by 1.6–1.8-fold, relative to wild-type (Figure 4B; Table S1). Interestingly, there was not as great an increase as might have been expected from the average number of nuclei in each wild-type fast muscle fibre (approximately 2.7 and 3.2 at 32 and 48 h. p. f., respectively; [20]). Staining mutant embryos with acridine orange did not reveal any increase in apoptosis relative to wild-type (unpublished data). In addition, we did not observe any rounded, unelongated, unfused myoblasts expressing fMyHC in either mutant or wild-type embryos (Figure 3B), suggesting that all somitic fast muscle myoblasts had undergone differentiation. Between 32 and 48 h. p. f., myotome muscle fibre number increased by a similar proportion in both mutant and wild-type embryos (Figure 4B). In contrast, the number of nuclei within each mutant myotome was decreased compared to wild-type embryos (Figure 4C; Table S2), suggesting that myoblast proliferation is limited in both mutants. In other words, growth of mutant, mononucleate fast muscle myotome requires less myocytes than the equivalent amount of growth of wild-type, syncytial fast muscle myotome. Taken together, these results reveal that the majority of fast muscle myoblasts could elongate and form functional mononuclear muscle fibres, resulting in an overabundance of fast muscle (Figure S1). This suggests that axial fast muscle precursors are not divided into distinct subpopulations.

### Jamb and Jamc Physically Interact

Both *jamb* and *jamc* are expressed in fast muscle myoblasts during primary myogenesis (Figure 1) and loss-of-function of either gene results in a severe block in myocyte fusion (Figure 2), without overtly affecting any other aspect of axial muscle differentiation (Figure 3). Taken together, these results suggest that Jamb and Jamc are a receptor pair necessary for myocyte fusion.

**Figure 5:**
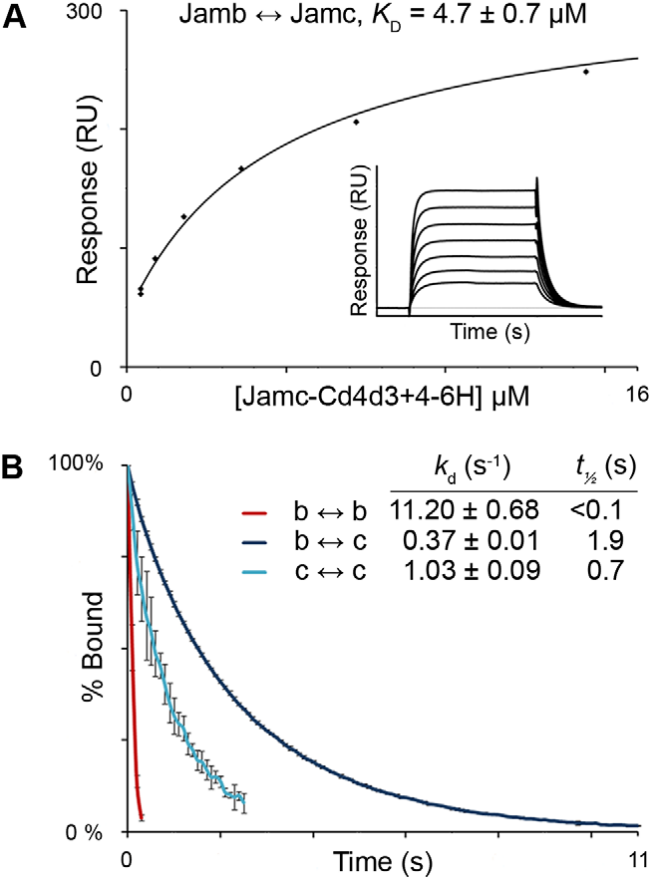
Jamb and Jamc physically interact. (A) Surface plasmon resonance experiments determine the equilibrium binding constant (*K*_D_) for the heterophilic interaction between Jamb and Jamc (inset: sensorgrams showing equilibrium has been reached). (B) Dissociation rate constants and half-lives of homophilic and heterophilic interactions between recombinant extracellular domains of Jamb and Jamc. Curves represent a fit to a first-order decay, indicating a 1∶1 stoichiometry of binding. Dissociation data are the mean of three analyte concentrations for each interaction tested; error bars represent standard deviation.

The mammalian orthologues of Jamb and Jamc are known to form both homophilic [41],[42] and heterophilic [43] interaction pairs. Our large-scale systematic protein interaction screen identified a heterophilic interaction between zebrafish Jamb and Jamc, but no homophilic binding was observed [33]. Homophilic interactions are known to be the main class of false negatives in the AVEXIS assay used in these screens [33], so to determine whether zebrafish Jamb and Jamc could interact homophilically and to quantify the relative biophysical binding parameters, we used soluble recombinant proteins and surface plasmon resonance. We found that both Jamb and Jamc were able to bind each other with an equilibrium binding constant typical of extracellular protein interactions between membrane-embedded cell surface receptors (*K*_D_≈4.7±0.7 µM, Figure 5A; [44]). To compare between all three possible interactions of Jamb and Jamc, we used dissociation phase data of binding experiments to calculate dissociation rate constants (Figure 5B). We took this approach because equilibrium measurements can be confounded by unreliable estimates of analyte activities, which are affected by homophilic interactions within the analyte. Dissociation rate constants are independent of analyte activity and can therefore be more appropriately compared. As expected from studies of the mammalian orthologues, both proteins could also self-associate, but with a much weaker interaction strength than that of the heterophilic interaction (Figure 5B). All dissociation curves fitted a first-order decay equation well, suggesting a 1-to-1 binding mechanism. These experiments show that while zebrafish Jamb and Jamc are indeed able to form homodimers, the heterophilic interaction between them is significantly stronger.

### The Interaction Between Jamb and Jamc Is Essential for Fusion In Vivo

Having established that both proteins could physically interact (Figure 5) and that *jamb* and *jamc* are co-expressed by myoblasts in wild-type (Figure 1) and mutant embryos (Figure S2), we used cellular transplantation experiments to determine the mechanism of binding between Jamb and Jamc for myocyte fusion in vivo.

Firstly, to demonstrate that *jamb^HU3319^* mutant myocytes are unable to fuse to each other, as observed in the mutant phenotype (Figure 2), we transplanted fluorescent dextran-labelled cells from *jamb^HU3319^* donors into *jamb^HU3319^* hosts and counted the number of labelled mononucleated or multinucleated fibres at 48 h. p. f. (Table 1). As expected, only 5% of myocytes derived from transplanted donor cells were able to fuse to mutant host myocytes, showing that expression of Jamc is unable to compensate for the loss of Jamb.

**Figure 6:**
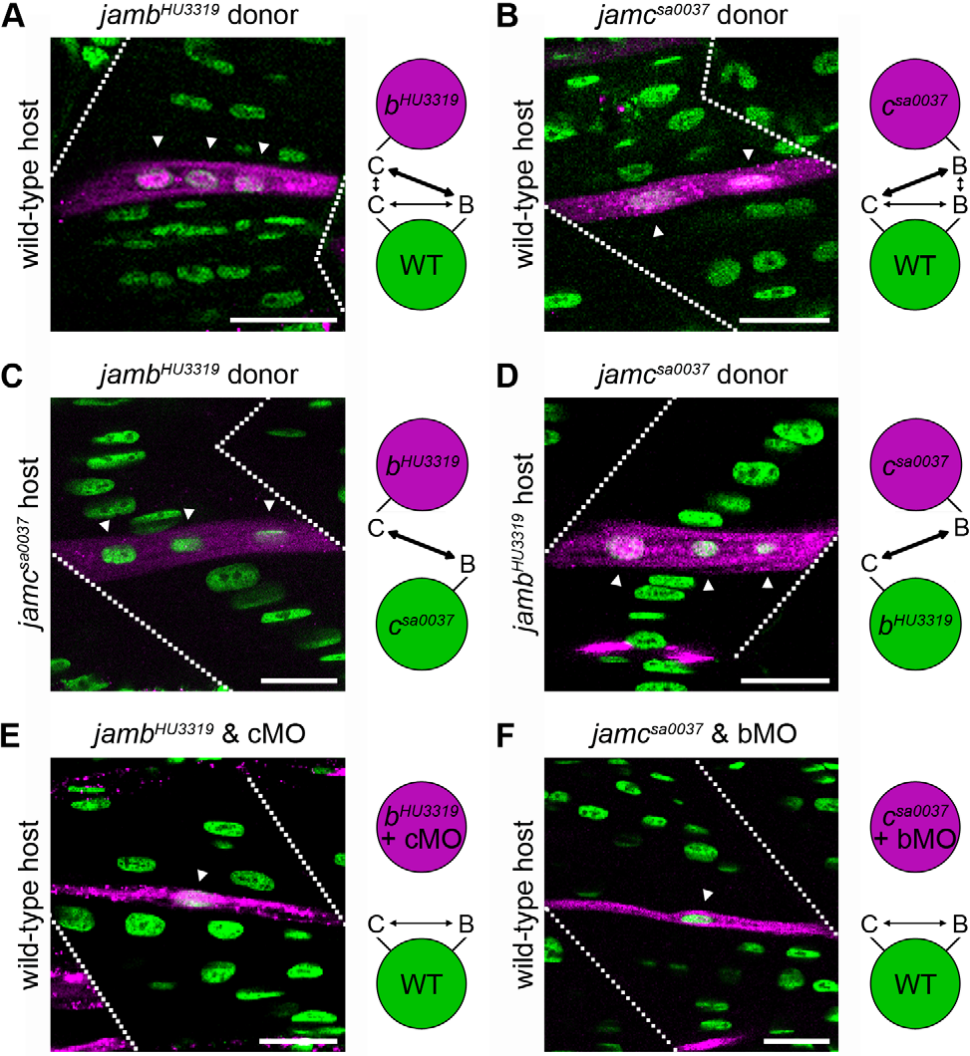
Interaction between *jamb* and *jamc* is necessary and required in trans for myocyte fusion. (A–D) Fluorescent dextran-labelled cells (magenta) from *jamb^HU3319^* and *jamc^sa0037^* donors can form multinucleate fibres with wild-type (A, B), *jamc^sa0037^* (C), or *jamb^HU3319^* (D) host cells, respectively. (E, F) Transplanted cells from doubly-deficient donors fail to fuse with wild-type host cells, suggesting both proteins are required and interact in trans. Confocal microscopy images from 48 h. p. f. embryos; anterior left. Schematics illustrate potential binding of Jamb and Jamc between donor (magenta) and host (green) cells in each experiment. bMO, cMO indicate *jamb* or *jamc* translation-blocking morpholino-injected donor embryos. Dotted lines indicate the position of myotome boundaries; arrowheads indicate nuclei within labelled fibres. Nuclei stained with DAPI (green). Scale bars represent 20 µm.

To establish if myocytes lacking *jamb* are nevertheless competent for fusion, we transplanted cells from *jamb^HU3319^* donors into wild-type hosts, and vice versa (Figure 6A and Table 1). When transplanted into wild-type hosts, 93% of *jamb^HU3319^* mutant cells could form multinucleate fibres, suggesting they are able to fuse with wild-type myocytes. Similarly, 95% of wild-type myocytes were able to form multinucleate fibres when transplanted into *jamb^HU3319^* hosts (Table 1). These results demonstrate that Jamb acts non-cell-autonomously, and that Jamb and Jamc need to be expressed by neighbouring cells for fusion to occur.

To determine if the Jamb and Jamc interaction between cells is necessary for fusion, we tested the prediction that transplanted *jamb^HU3319^* mutant cells (that could nevertheless express wild-type Jamc) would be able to fuse to *jamc^sa0037^* hosts (that could express wild-type Jamb). We observed that 96% of *jamb^HU3319^* mutant donor cells were able to fuse to *jamc^sa0037^* mutant host cells (Figure 6C, Table 1). The cellular complementation between *jamb* and *jamc* mutant myocytes demonstrates that Jamb and Jamc must interact as a heterophilic pair on neighbouring cells and do not act as independent homophilic receptors.

To show that the interaction between Jamb and Jamc proteins was necessary for fusion and did not require any additional factors, donor cells that were deficient in both Jamb and Jamc (*jamb^HU3319^* embryos injected with a *jamc*-targeted morpholino) were transplanted into wild-type hosts (Figure 6E; Table 1). Most doubly-deficient donor cells (88%) could not fuse with wild-type host cells, demonstrating that expression of either Jamb or Jamc is essential for a myocyte to be competent for fusion. Doubly-deficient embryos are indistinguishable from *jamb^HU3319^* and *jamc^sa0037^* mutant embryos, suggesting no further phenotypic enhancement from combined knockdown of both proteins (Figure S3).

We repeated each of the transplant experiments described above, except we used *jamc^sa0037^* mutants as donors to establish if myocytes from both mutants behaved similarly. As with *jamb^HU3319^* transplants, we observed that *jamc^sa0037^* donor cells were unable to fuse to *jamc^sa0037^* host cells (Table 1), showing that Jamb is unable to rescue the loss of Jamc; Jamb and Jamc are not redundant. Wild-type donor cells were able to fuse to *jamc^sa0037^* host cells and vice versa (Figure 6B, Table 1), demonstrating that *jamc* mutant myocytes are competent for fusion and suggesting that Jamb and Jamc need to be expressed by neighbouring cells for fusion to occur between them. In addition, *jamc^sa0037^* donor cells were able to complement *jamb^HU3319^* host cells and fuse with wild-type efficiency (95%, Figure 6D, Table 1). This reinforces the conclusion that Jamb and Jamc must interact as a heterophilic pair between adjacent cells for fusion to occur. Finally, doubly deficient cells (*jamc^sa0037^* embryos injected with a *jamb*-targeted morpholino; Figure S3) were unable to fuse to wild-type host myocytes (Figure 6F, Table 1), further demonstrating that no other factor is interacting with either Jamb or Jamc. Interestingly, *jamc^sa0037^* mutant cells transplanted into a wild-type host fused less efficiently than into a *jamb^HU3319^* host, suggesting that homophilic Jamb interactions between donor and host myocytes could inhibit fusion in the absence of Jamc on the donor cell (compare *jamc^sa0037^* donor, wild-type host and *jamc^sa0037^* donor, *jamb^HU3319^* host; Table 1, Figure 6B and D). Taken together, these data show that the physical interaction between Jamb-Jamc is required between neighbouring cells for myocyte fusion to occur in vivo.

### *jamc* Is Ectopically Expressed in *prdm1a* Mutant Slow Muscle Cells That Fuse Inappropriately

The overabundance of fast muscle fibres in the absence of myocyte fusion suggested that the regulation of *jamb* and *jamc* might play an important role in the control of muscle patterning and development.

Slow-twitch muscle myocytes do not undergo fusion during primary myogenesis [40]. However, in zebrafish embryos mutant for the transcriptional repressor *prdm1a*, the premigratory progenitors of slow-twitch muscle [39] express fast muscle-specific genes [45] and inappropriately fuse with the neighbouring fast muscle myocytes, resulting in the absence of slow muscle fibres [40]. These observations suggest that *prdm1a* mutant adaxial cells must ectopically express critical cell surface proteins necessary for myocyte fusion.

**Figure 7:**
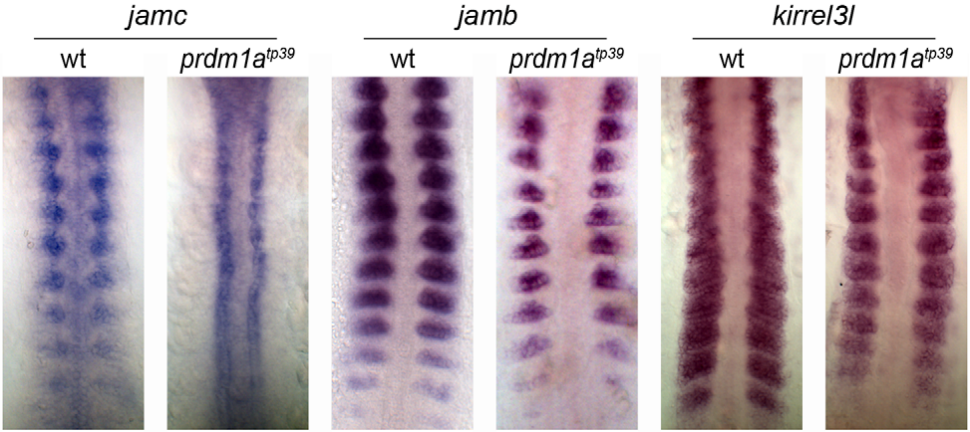
*jamc* expression is regulated by *prdm1a*. Wholemount in situ hybridisation against *jamc* (left panels), *jamb* (middle panels), and *kirrel3l* (right panels) shows that *jamc* is ectopically expressed in premigratory slow muscle precursors (adaxial cells) of *prdm1a^tp39^* mutants that later inappropriately fuse to fast muscle myocytes. In contrast, *jamb* and *kirrel3l* are expressed in fast muscle myoblasts in both wild-type and *prdm1a^tp39^* mutant embryos. Flatmounted embryos at 10–13 somite stage; anterior top.

To test if either *jamb*, *jamc*, or *kirrel3l* [22] are ectopically expressed by *prdm1a* mutant adaxial cells, we performed wholemount in situ hybridization using riboprobes for each gene. We observed that *jamc* is misexpressed in the adaxial cells of *prdm1a^tp39^* embryos, but that *jamb* and *kirrel3l* are not (Figure 7). All three genes are expressed in fast muscle myoblasts of *prdm1a^tp39^* mutant embryos, as expected (Figure 7, Figure S4). These results suggest that misregulation of *jamc* permits ectopic fusion of mutant slow muscle precursors with neighbouring *jamb*-expressing fast muscle myocytes; in wild-type embryos, *prdm1a* represses *jamc* in adaxial cells to prevent this occurring. This also implies that a heterophilic interaction of Jamb and Jamc between mutant slow muscle cells and fast muscle myocytes is necessary for ectopic fusion events to occur. Finally, these data also suggest that transcriptional regulation of *jamc* triggers fast muscle myocyte fusion events in vertebrate musculature.

To determine if expression of *jamc* alone is sufficient to cause fusion events between slow muscle cells and fast muscle myocytes, we attempted to ectopically express *jamc* in wild-type slow muscle by microinjecting transgenic embryos containing a slow muscle marker, *Tg(smyhc1::egfp)^i104^* [46], with full-length, capped *jamc* mRNA or a plasmid containing full-length *jamc*. We did not observe any ectopic fusion events with slow muscle cells in injected embryos (unpublished data), suggesting that other components, presumably also regulated by *prdm1a*, are necessary for fusion. Furthermore, we tested whether or not interaction between Jamb and Jamc is sufficient to drive fusion in heterologous cells, as described for the fusogen EFF-1 [47], by mixing HEK293E cells transfected with plasmids containing either full-length *jamb* or *jamc*. No fusion events were observed in mixed cultures of *jamb*- and *jamc*-transfected cells, or control cultures containing only *jamb*- or *jamc*-transfected cells (unpublished data). These results suggest that the context of Jamb and Jamc binding determines whether or not the interacting cells will fuse.

## Discussion

Using a combination of quantitative biochemistry, mutant zebrafish, and cell transplantation experiments, we have shown that we have identified a heterophilic interaction between a cell surface receptor pair that is essential in vivo for myocyte fusion in a vertebrate. This discovery has important implications for the molecular mechanism, regulation, and evolution of cellular fusion in the context of myogenesis.

The identification of Kirrel3l as a functional orthologue of Kirre/Rst in zebrafish and of other key intracellular effectors [19]–[23] suggests conservation of the important signalling pathways for the process of myocyte fusion between vertebrates and invertebrates. Our discovery of Jamb and Jamc as a new deuterostome-restricted [31] receptor pair that is essential for fusion in the zebrafish axial musculature raises the possibility of vertebrate-specific adaptations of the components and the regulation of muscle development in vertebrates. For example, our results suggest that this interaction is independent of Kirrel3l, suggesting that multiple recognition steps between vertebrate myocytes are required for fusion.

During differentiation, myocytes make a fundamental decision between founding a new muscle fibre or fusing to an existing one. In chicken and mouse embryos this decision seems to be temporally controlled: primary myocytes form an array of elongated mononucleate fibres, to which later differentiating myocytes fuse [48],[49]. In the larval body wall of *Drosophila*, this decision is controlled by early specification of myoblasts into two distinct subtypes, ultimately defining the number of muscle fibres formed [7],[12]–[16],[50]. Our results show that in the absence of fusion in the zebrafish axial musculature, the number of fast-twitch muscle fibres almost doubles, suggesting that the fast muscle precursors are not divided into defined subpopulations, but that each myocyte is capable of founding a fibre if it does not fuse to an existing one. The co-expression of these essential receptors in all fast muscle myoblasts adds to the suggestion that the precursors are not restricted to specific fates. In addition, our transplantation experiments did not reveal any functional subdivision of myocytes; approximately 95% of *jamb* or *jamc* mutant donor myocytes were able to undergo fusion with *jamc* or *jamb* host myocytes, respectively. The dynamic nature of *jamc* expression in fast muscle myoblasts and repression in slow muscle precursors suggest that regulation of *jamc* plays a fundamental role in the patterning of muscles through the timing of fusion events, rather than specification. Furthermore, other elements of terminal differentiation, such as elongation and sarcomerogenesis, seem to be independent of the process of fusion.

Our results suggest that the interaction between Jamb and Jamc expressed by neighbouring cells is essential for fusion. These cell surface receptors likely mediate an initial recognition and adhesion event similar to that of the cell surface receptors Kirre and Sns in *Drosophila*. It is unlikely that the interaction between Jamb and Jamc is sufficient for fusion because both proteins are known to be expressed and interact in other tissues that do not normally undergo fusion, such as the vascular endothelium [51]. Jamb and Jamc permit cellular recognition and adhesion, but do not cause fusion when expressed in heterologous cells such as CHO [52], MDCK [51], or HEK293E (unpublished data), unlike EFF-1, a known fusogen in *C. elegans* that causes spontaneous fusion between Sf9 insect cells transfected with membrane-bound splice variants [47]. In addition, ectopically expressing either Jamb or Jamc in zebrafish slow muscle cells did not result in inappropriate fusion with fast muscle precursors (unpublished data). We hypothesise that the biological context of Jamb and Jamc binding determines the productive output of that interaction; for example, cellular fusion or tight junction formation. Interaction between Kirre and Sns is thought to be the initiation event for the formation of a crucial adhesion and signalling complex between a founder cell and a fusion-competent myoblast, termed FuRMAS [9]. Both Kirre and Sns are thought to be involved in localising important signalling components to this complex, such as Rols7 and Mbc, in order to build and maintain a complex branched F-actin structure necessary for fusion [11]. Similarly, Jam-B and Jam-C are known to be involved in forming tight junctions between cells and localising other proteins such as ZO-1 to those sites [27]–[29]. A specialised fusion structure has not been reported or characterised in vertebrates to date, but Jamb and Jamc may form part of a similar complex that defines the site of fusion between myocytes.

A conserved role for JAM-B and JAM-C in myocyte fusion in other vertebrate organisms is an important focus for our future research. In support of this hypothesis, both genes have been shown to be expressed in developing skeletal muscle of mouse [53],[54] and human embryos [55]. Knockout mice models have been generated for both genes and studied in the context of fertility [30], immunity [56],[57], cardiac development [58], neurobiology [59], and stem cell biology [60]. Two independent *Jam-C* ^−/−^ models have been reported with high perinatal mortality [30],[57]; approximately two-thirds of mutant pups die within 48 h after birth and are described as cyanotic and gasping [57],[58]. The formation, structure, and integrity of the diaphragm has not been studied in these mice. Surviving *Jam-C* mutant mice also exhibit significant growth retardation starting from the second week of perinatal development [56],[58], megaeosophagus [56]–[58], weaker forepaw grip strength [59], and “jitteriness” [58]. These characteristics could conceivably be a result of underlying muscle defects. Mice deficient in *Jam-B* display no overt phenotype [53],[60] although skeletal muscle development and growth have not been specifically examined in detail.

We believe that identification of the critical function of *jamb* and *jamc* in zebrafish myocyte fusion presents us with an opportunity to better understand myogenesis in higher vertebrates and cellular fusion in other biological contexts. A molecular explanation of the intercellular recognition processes that are necessary for fusion in, for example, placenta formation and sperm-egg interactions remains incomplete. The identification of Jamb and Jamc as an in vivo validated receptor-ligand pair required for cellular fusion in vertebrates may now provide impetus to shed more light on these biological processes.

## Materials and Methods

### Zebrafish Husbandry, Embryo Culture, and Fixation

Zebrafish mutants carrying alleles *jamb^HU3319^* and *jamc^sa0037^* were obtained from the Hubrecht laboratory and Wellcome Trust Sanger Institute Zebrafish Mutant Resource and maintained according to standard fish husbandry conditions and UK Home Office and Institute regulations and guidelines. Both *jamb* and *jamc* mutant lines were homozygous viable and fertile in our aquarium, but did not thrive. Embryos were fixed in either 4% paraformaldehyde or, for EB165 immunohistochemistry, in methanol.

### Nomenclature and Accession Numbers

We refer to the zebrafish homologues of *JAM-B* and *JAM-C* as *jamb* and *jamc*, respectively, for the sake of clarity and consistency with other recent literature concerning the *JAM* family [26]. The official symbols and accession/reference numbers are as follows: *jamb* (official symbol *jam2a*) - Entrez gene: 100005261; *jamc* (official symbol *jam3b*) - Entrez Gene: 569217.

### Protein Production, Purification, and Surface Plasmon Resonance

The extracellular domain of Jamb or Jamc were expressed as a soluble fusion protein with rat Cd4 domains 3 and 4 and either a 6-histidine (Cd4d3+4-6H) or an enzymatically biotinylatable peptide (Cd4d3+4-bio) C-terminal tag. These were purified and used in surface plasmon resonance experiments, essentially as previously described [33]. The activity of the Jamc analyte used in binding experiments cannot be accurately determined, as Jamc is capable of homophilic association. Dissociation rate constants (*k*_d_), which are not confounded by analyte activities (and can therefore be directly compared), were calculated by averaging the dissociation phase of three different concentrations of purified Jamc-Cd4d3+4-6H or Jamb-Cd4d3+4-6H protein and fitting a simple first-order decay curve. Fits to the data were good, suggesting a 1∶1 stoichiometry of binding. Half lives (*t*_½_) were calculated by *t_½_* = *ln* 2/*k_d_*.

### Wholemount In Situ Hybridisation and Immunohistochemistry

Wholemount in situ hybridisations using digoxygenin-labelled riboprobes were performed using standard protocols [61]. Riboprobe templates were generated from plasmids containing the extracellular domain of *jamb*, *jamc*, or *kirrel3l*.

Wholemount immunohistochemistry was performed according to standard methods, using mouse monoclonal antibodies F59, EB165 (1∶200; Developmental Studies Hybridoma Bank) and anti-mouse IgG, Alexa-488- or Alexa-568-conjugated secondary antibodies (1∶5,000; Molecular Probes). Embryos were mounted in Slowfade Gold with DAPI (Molecular Probes) and/or treated with Alexa-488-conjugated phalloidin (1∶40; Cambrex Biosciences).

### Labelling Cell Membranes with mRFP

Capped membrane-targeted red fluorescent protein mRNA was transcribed from a linearised plasmid [62] using the mMessage mMachine kit (Ambion) and SP6 polymerase. 1–2 cell stage embryos were microinjected with approximately 4 nl of mRNA (∼25 ng/µl) diluted in sterile water, 0.1% phenol red (Sigma-Aldrich), fixed with 4% paraformaldehyde, and observed by confocal microscopy. Optical cross-sections of fixed, 48 h. p. f. mRFP-labelled embryos were computed from z-stacks collected from myotomes 10–15 in each embryo, using Leica Application Suite Advanced Fluorescence software (LAS AF; Leica Microsystems). Fibres were manually counted in each cross-section; superficial slow muscle fibres were excluded from analysis. Estimation of nuclei was determined by *mf_h_n_h_*+(*1−m*)*f_h_*, where *m* is the fraction of multinucleated fibres (quantified in same donor into same host genotype transplant controls; Table S1), *f_h_* is the number of fibres, *n_h_* is the average number of nuclei per fibre reported [20], and *h* is the developmental stage in h. p. f.

### Morpholino Treatment

1- and 2-cell stage embryos were injected with approximately 4 nl of translation blocking morpholinos (∼200 µM, 5–7.5 ng per embryo) diluted in sterile water with 0.1% phenol red. Translation blocking morpholino sequences were as follows: *jamb*: GCA CAC CAG CAT TTT CTC CAC AGT G; *jamc*: TTA ACG CCA TCT TGG AGT CGG TGA A.

### Cell Transplants

Transplants were performed essentially as described [63]. Briefly, 1–2-cell stage donor embryos were injected with lysine-fixable fluorescein or rhodamine labelled dextran (10,000 kDa, 1% in sterile water; Molecular Probes). Fluorescently labelled donor cells were transplanted into the marginal cells of unlabelled host embryos between high/sphere to ∼30% epiboly stages. Transplanted embryos were maintained in embryo media supplemented with penicillin (50 U/ml) and streptomycin (50 µg/ml), fixed in 4% paraformaldehyde at 48 h. p. f., and analysed by confocal microscopy.

### Image Acquisition and Processing

Confocal microscopy images were collected using a Leica SP5/DM6000 confocal microscope and LAS AF software. Wholemount in situ hybridisation images were obtained using a Zeiss Imager M1 microscope, Zeiss AxioCam Hrc camera, and Zeiss AxioVision software. Entire images were adjusted for contrast, brightness, dynamic range, and resampled to a standardised resolution (300 d. p. i.) using Adobe Photoshop CS2.

### Statistical Analysis

Statistical significance between wild-type and mutant fibre counts and nuclei estimates were determined by one-tailed Student's *t* test, modified to take unequal sample size and variance into account. The number of embryos is presented in Table S1.

## Supporting Information

Figure S1 Model of fast muscle development in *jam* mutant and wild-type embryos. Each panel presents a schematic of a single somite (so), notochord (nc), and adaxial cell (ad) or migrating slow muscle fibres (sm) as viewed dorsally, anterior left, at different stages during somitogenesis; latest stage to the right. In wild-type embryos, fast-twitch myoblasts (fMBs) express *jamb* (blue). At approximately 10–13 somites stage, medio-posterior myoblasts begin to express *jamc*, in addition to *jamb* (red), and differentiate (upper left panel). Other myocytes are able to fuse to the *jamb*, *jamc* expressing myocytes once fully elongated (white arrows, upper middle panel) resulting in multinucleated muscle fibres (upper right panel; nuclei in dark red). This process continues medio-laterally, as slow muscle fibres (sm) migrate to a superficial position (yellow arrow), until all primary somitic fast-twitch myoblasts have fused together to form the fast muscle myotome. Future growth of the myotome requires proliferation of the external cell layer (yellow cells)—myoblasts that are initially within the anterior border of the early somite (ABCs, anterior border cells). In *jam* mutant embryos, *jamb* and *jamc* are expressed normally (lower left panel). In contrast to wild-type, *jamb^HU3319^* or *jamc^sa0037^* myocytes are unable to undergo fusion (lower middle panel) and instead differentiate to form mononucleate fibres (lower right panel), nearly doubling the number of fast muscle fibres.
(TIF)

Figure S2 Expression of *jamb* and *jamc* in *jamb^HU3319^* and *jamc^sa0037^* mutant embryos. In situ hybridisation of *jamb* (left two panels) and *jamc* (right two panels) riboprobes to *jamb^HU3319^* and *jamc^sa0037^* embryos at 17–18 somites stage; anterior top. Both genes are expressed in fast muscle myoblasts in both *jamb^HU3319^* and *jamc^sa0037^* mutants as observed in wild-type embryos.
(TIF)

Figure S3 Combined knockdown of *jamb* and *jamc* does not result in a synthetic myogenesis phenotype. (A, B) Antisense morpholino oligonucleotide knockdown of expression of *jamc* in *jamb^HU3319^* embryos (A, right) or *jamb* in *jamc^sa0037^* embryos (B, right) does not result in any further disruption of myogenesis than that observed in *jamb^HU3319^* (A, left) or *jamc^sa0037^* (B, left) at 48 h. p. f., suggesting no synthetic effect of combined knockdown of both genes. Single confocal microscopy images of myotomes 12–13 in 48 h. p. f. embryos, stained for F-actin (cyan) and nuclei (red). Anterior left; scale bars represent 50 µm.
(TIF)

Figure S4 *jamc* expression in fast muscle myoblasts and adaxial cells of *prdm1a^tp39^* mutants. *jamc* is expressed in fast muscle myoblasts (open arrowheads) and ectopically expressed in premigratory slow muscle precursors (adaxial cells; closed arrowheads) of *prdm1a^tp39^* mutant embryos (bottom). Flatmounted wild-type sibling (top) and *prdm1a^tp39^* mutant embryos (bottom) at 18–20 somites stage, hybridised to *jamc*; anterior left.
(TIF)

Table S1 Average number of fast muscle fibres per myotome in wild-type and mutant embryos at different developmental stages. Values presented as mean ± SD; *n*, number of embryos tested; n.a., not applicable as fibres have not elongated. ^†^Significantly different from wild-type, *p*≤0.001. ^‡^Significantly different from *jamb^HU3319^*, *p*≤0.01. One-tailed *t* test, modified to account for unequal sample sizes and sample variance.
(DOC)

Table S2 Average number of nuclei per myotome, calculated from number of fast muscle fibres per myotome in wild-type and mutant embryos at different developmental stages, taking the fraction of multinucleated fibres into account (Table 1). Values presented as mean ± SD, number of embryos tested as in Table S1. *Values from (Moore et al., 2007) [20]. ^†^Significantly different from wild-type, p≤0.001. ^‡^Significantly different from *jamb^HU3319^*, p≤0.01. One-tailed *t* test, modified to account for unequal sample sizes and sample variance.
(DOC)

## Abbreviations

AVEXIS: avidity based extracellular interaction screen
CHO: chinese hamster ovary cells
FCM: fusion competent myoblast
fMyHC: fast isoform myosin heavy chain
FuRMAS: fusion-restricted myogenic-adhesive structure
h. p. f.: hours post-fertilisation
HEK293E: human embryonic kidney 293 cells
MDCK: Madin-Darby canine kidney epithelial cells
MO: morpholino
mRFP: membrane-targeted red fluorescent protein
sMyHC: slow isoform myosin heavy chain
UTR: untranslated region
