# Supplementary figures and images for: Jamb and Jamc Are Essential for Vertebrate Myocyte Fusion
Source: PLoS Biol. 2011 Dec 13;9(12):e1001216. doi: 10.1371/journal.pbio.1001216 (PMC3236736; doi:10.1371/journal.pbio.1001216)

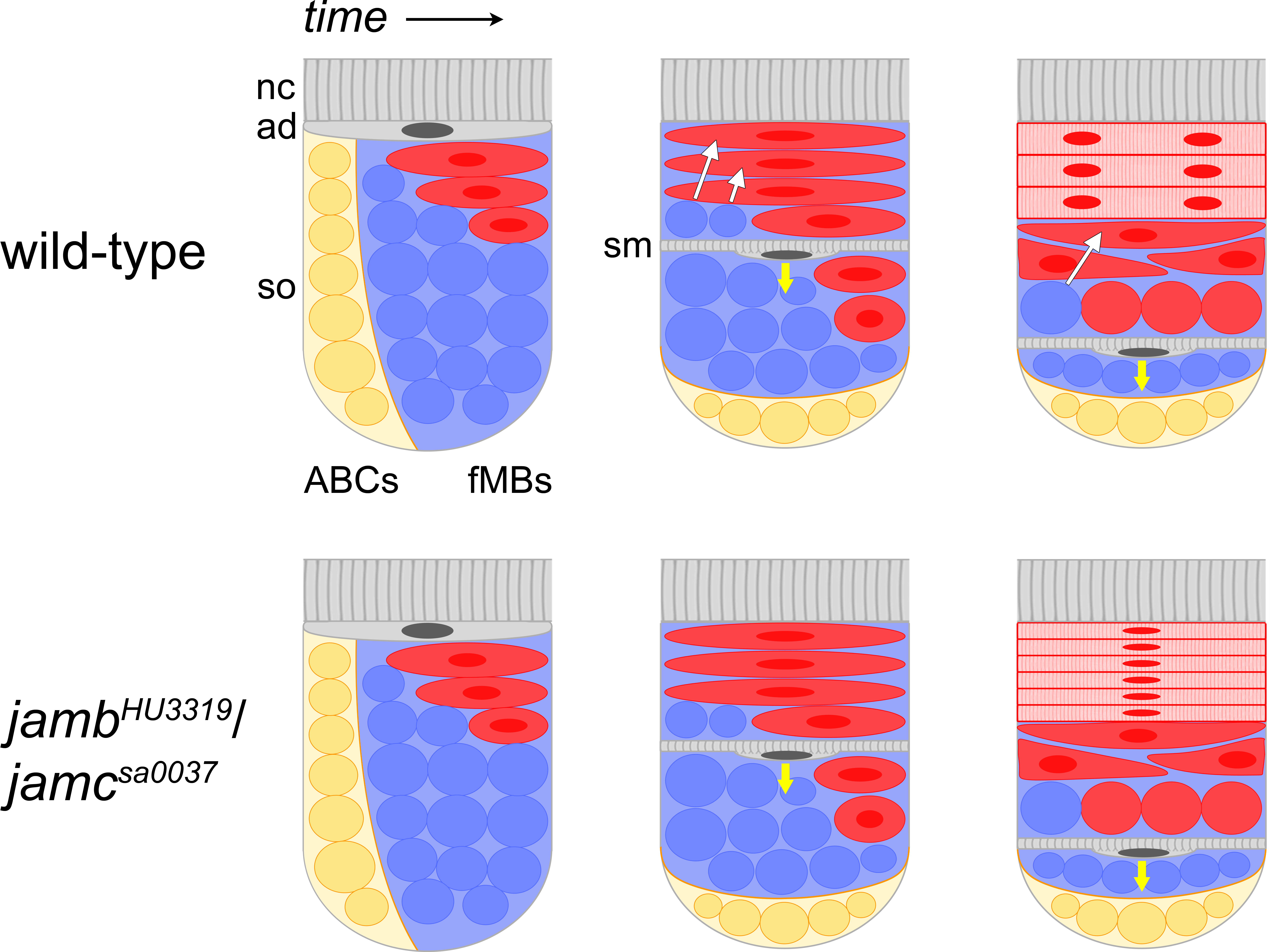

Supplement: Figure S1 — Model of fast muscle development in jam mutant and wild-type embryos. Each panel presents a schematic of a single somite (so), notochord (nc), and adaxial cell (ad) or migrating slow muscle fibres (sm) as viewed dorsally, anterior left, at different stages during somitogenesis; latest stage to the right. In wild-type embryos, fast-twitch myoblasts (fMBs) express jamb (blue). At approximately 10–13 somites stage, medio-posterior myoblasts begin to express jamc, in addition to jamb (red), and differentiate (upper left panel). Other myocytes are able to fuse to the jamb, jamc expressing myocytes once fully elongated (white arrows, upper middle panel) resulting in multinucleated muscle fibres (upper right panel; nuclei in dark red). This process continues medio-laterally, as slow muscle fibres (sm) migrate to a superficial position (yellow arrow), until all primary somitic fast-twitch myoblasts have fused together to form the fast muscle myotome. Future growth of the myotome requires proliferation of the external cell layer (yellow cells)—myoblasts that are initially within the anterior border of the early somite (ABCs, anterior border cells). In jam mutant embryos, jamb and jamc are expressed normally (lower left panel). In contrast to wild-type, jambHU3319 or jamcsa0037 myocytes are unable to undergo fusion (lower middle panel) and instead differentiate to form mononucleate fibres (lower right panel), nearly doubling the number of fast muscle fibres. (TIF) [file pbio.1001216.s001.tif]

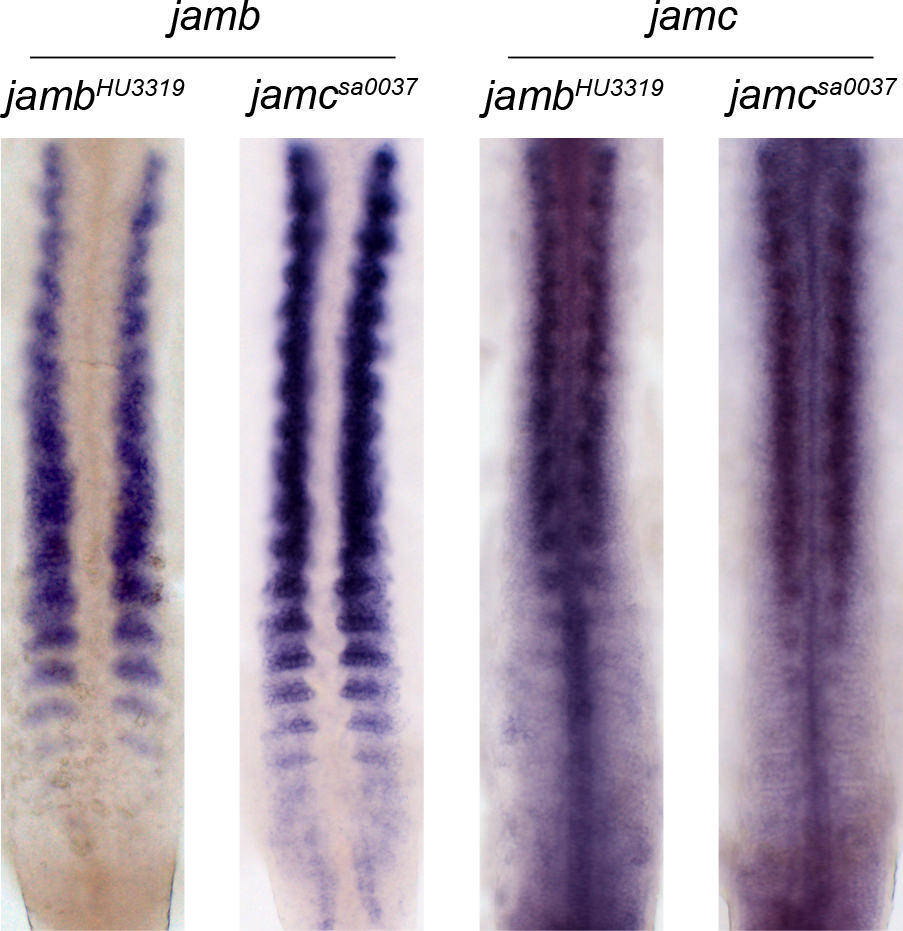

Supplement: Figure S2 — Expression of jamb and jamc in jambHU3319 and jamcsa0037 mutant embryos. In situ hybridisation of jamb (left two panels) and jamc (right two panels) riboprobes to jambHU3319 and jamcsa0037 embryos at 17–18 somites stage; anterior top. Both genes are expressed in fast muscle myoblasts in both jambHU3319 and jamcsa0037 mutants as observed in wild-type embryos. (TIF) [file pbio.1001216.s002.tif]

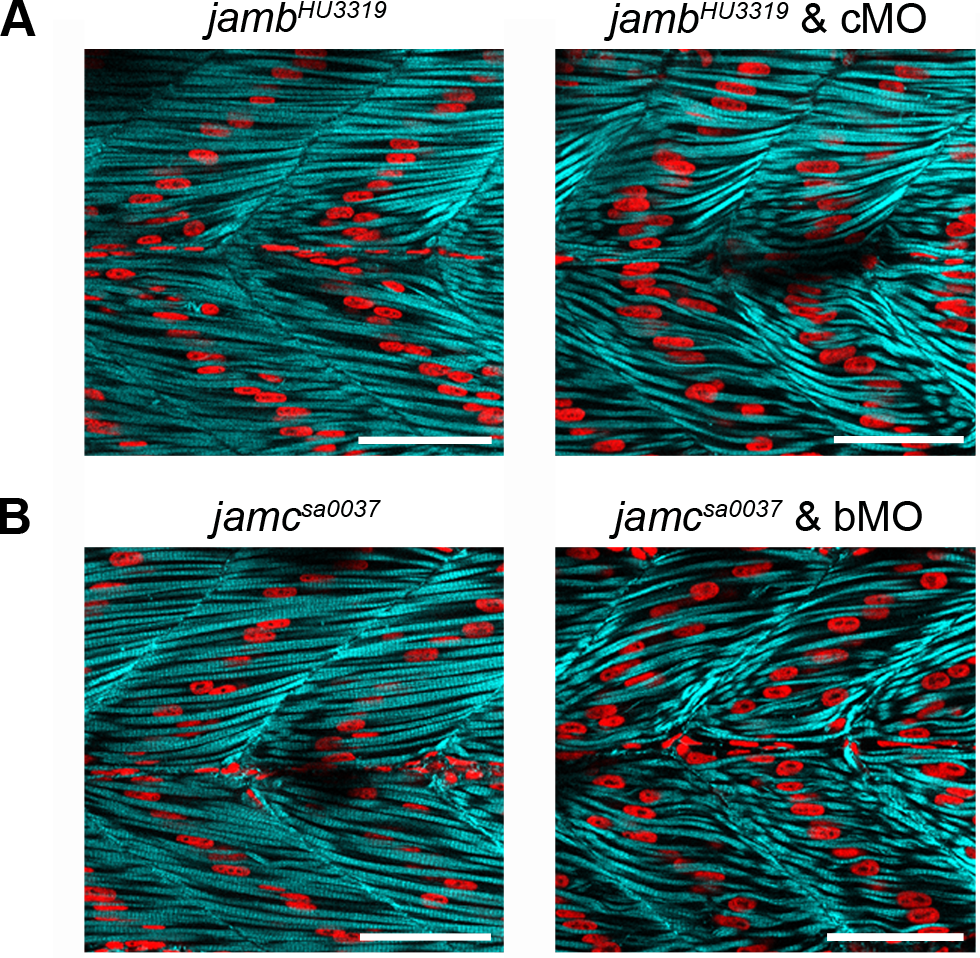

Supplement: Figure S3 — Combined knockdown of jamb and jamc does not result in a synthetic myogenesis phenotype. (A, B) Antisense morpholino oligonucleotide knockdown of expression of jamc in jambHU3319 embryos (A, right) or jamb in jamcsa0037 embryos (B, right) does not result in any further disruption of myogenesis than that observed in jambHU3319 (A, left) or jamcsa0037 (B, left) at 48 h. p. f., suggesting no synthetic effect of combined knockdown of both genes. Single confocal microscopy images of myotomes 12–13 in 48 h. p. f. embryos, stained for F-actin (cyan) and nuclei (red). Anterior left; scale bars represent 50 µm. (TIF) [file pbio.1001216.s003.tif]

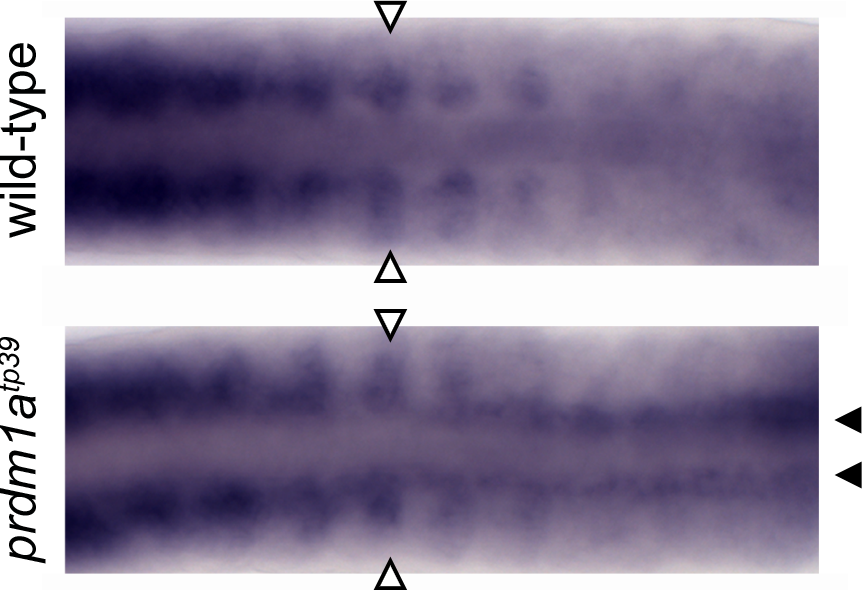

Supplement: Figure S4 — jamc expression in fast muscle myoblasts and adaxial cells of prdm1atp39 mutants. jamc is expressed in fast muscle myoblasts (open arrowheads) and ectopically expressed in premigratory slow muscle precursors (adaxial cells; closed arrowheads) of prdm1atp39 mutant embryos (bottom). Flatmounted wild-type sibling (top) and prdm1atp39 mutant embryos (bottom) at 18–20 somites stage, hybridised to jamc; anterior left. (TIF) [file pbio.1001216.s004.tif]
