# Supplementary material for: Jamb and Jamc Are Essential for Vertebrate Myocyte Fusion
Source: PLoS Biol. 2011 Dec 13;9(12):e1001216. doi: 10.1371/journal.pbio.1001216 (PMC3236736; doi:10.1371/journal.pbio.1001216)
Supplement: Table S1 — Average number of fast muscle fibres per myotome in wild-type and mutant embryos at different developmental stages. Values presented as mean ± SD; n, number of embryos tested; n.a., not applicable as fibres have not elongated. †Significantly different from wild-type, p≤0.001. ‡Significantly different from jambHU3319, p≤0.01. One-tailed t test, modified to account for unequal sample sizes and sample variance. (DOC) [file pbio.1001216.s005.doc]

| Supplemental Table 1. Quantification of fast muscle fibres per myotome in wild-type, *jamb^hu3319^* and *jamc^sa0037^* embryos. | | | | | | | | |
| --- | --- | --- | --- | --- | --- | --- | --- | --- |
| Time  (h. p. f.) | Genotype | | | | | | | |
|  | wild-type (wt) | | *jamb^HU3319^* | | | *jamc^sa0037^* | | |
|  | no. fibres | n | no. fibres | ratio to wt | n | no. fibres | ratio to wt | n |
| 24 | 94 ± 12 | 12 | n. a. | | | n. a. | | |
| 32 | 108 ± 11 | 10 | 203 ± 16^†^ | 1.9 | 6 | 179 ± 13^†‡^ | 1.7 | 5 |
| 48 | 159 ± 17 | 8 | 290 ± 20^†^ | 1.8 | 11 | 258 ± 9^†‡^ | 1.6 | 6 |
| Average number of fast muscle fibres per myotome in wild-type and mutant embryos at different developmental stages. Values presented as mean ± S. D., n = number of embryos tested. n. a. = not applicable as fibres have not elongated. ^†^Significantly different from wild-type, p ≤ 0.001. ^‡^Significantly different from *jamb^HU3319^*, p ≤ 0.01. One-tailed t-test, modified to account for unequal sample sizes and sample variance. | | | | | | | | |
