# Supplementary material for: Jamb and Jamc Are Essential for Vertebrate Myocyte Fusion
Source: PLoS Biol. 2011 Dec 13;9(12):e1001216. doi: 10.1371/journal.pbio.1001216 (PMC3236736; doi:10.1371/journal.pbio.1001216)
Supplement: Table S2 — Average number of nuclei per myotome, calculated from number of fast muscle fibres per myotome in wild-type and mutant embryos at different developmental stages, taking the fraction of multinucleated fibres into account (Table 1). Values presented as mean ± SD, number of embryos tested as in Table S1. *Values from (Moore et al., 2007) [20]. †Significantly different from wild-type, p≤0.001. ‡Significantly different from jambHU3319, p≤0.01. One-tailed t test, modified to account for unequal sample sizes and sample variance. (DOC) [file pbio.1001216.s006.doc]

| **Supplemental Table 2.** Calculated number of nuclei per myotome in wild-type, *jamb^hu3319^* and *jamc^sa0037^* embryos. | | | | | | |
| --- | --- | --- | --- | --- | --- | --- |
| Time  (*h*; h. p. f.) | Average no. nuclei per fibre (*n_h_*)* | Genotype | | | | |
|  |  | wild-type (wt) | *jamb^HU3319^* | | *jamc^sa0037^* | |
|  |  | no. nuclei | no. nuclei | ratio to wt | no. nuclei | ratio to wt |
| 24 | 2.48 | 228 ± 29 | n. a. | | n. a. | |
| 32 | 2.70 | 286 ± 30 | 220 ± 17^†^ | 0.8 | 224 ± 16^†^ | 0.8 |
| 48 | 3.23 | 506 ± 56 | 322 ± 22^†^ | 0.6 | 345 ± 12^†‡^ | 0.7 |
| Average number of nuclei per myotome, calculated from number of fast muscle fibres per myotome in wild-type and mutant embryos at different developmental stages, taking the fraction of multinucleated fibres into account (Table 1). Values presented as mean ± S. D., number of embryos tested as in Supplemental Table 1. *Values from (Moore et al., 2007). ^†^Significantly different from wild-type, p ≤ 0.001. ^‡^Significantly different from *jamb^HU3319^*, p ≤ 0.01. One-tailed t-test, modified to account for unequal sample sizes and sample variance. | | | | | | |
